# Use of biological priors enhances understanding of genetic architecture and genomic prediction of complex traits within and between dairy cattle breeds

**DOI:** 10.1186/s12864-017-4004-z

**Published:** 2017-08-10

**Authors:** Lingzhao Fang, Goutam Sahana, Peipei Ma, Guosheng Su, Ying Yu, Shengli Zhang, Mogens Sandø Lund, Peter Sørensen

**Affiliations:** 1 Department of Molecular Biology and Genetics, Center for Quantitative Genetics and Genomics, Aarhus University, 8830 Tjele, Denmark; 2 Key Laboratory of Animal Genetics, Breeding and Reproduction, Ministry of Agriculture & National Engineering Laboratory for Animal Breeding, College of Animal Science and Technology, China Agricultural University, Beijing, 100193 China

**Keywords:** Genomic feature model, Genomic prediction, Genetic architecture, Gene ontology, Post-GWAS, Milk production, Mastitis, Dairy cattle

## Abstract

**Background:**

A better understanding of the genetic architecture underlying complex traits (e.g., the distribution of causal variants and their effects) may aid in the genomic prediction. Here, we hypothesized that the genomic variants of complex traits might be enriched in a subset of genomic regions defined by genes grouped on the basis of “Gene Ontology” (GO), and that incorporating this independent biological information into genomic prediction models might improve their predictive ability.

**Results:**

Four complex traits (i.e., milk, fat and protein yields, and mastitis) together with imputed sequence variants in Holstein (HOL) and Jersey (JER) cattle were analysed. We first carried out a post-GWAS analysis in a HOL training population to assess the degree of enrichment of the association signals in the gene regions defined by each GO term. We then extended the genomic best linear unbiased prediction model (GBLUP) to a genomic feature BLUP (GFBLUP) model, including an additional genomic effect quantifying the joint effect of a group of variants located in a genomic feature. The GBLUP model using a single random effect assumes that all genomic variants contribute to the genomic relationship equally, whereas GFBLUP attributes different weights to the individual genomic relationships in the prediction equation based on the estimated genomic parameters. Our results demonstrate that the immune-relevant GO terms were more associated with mastitis than milk production, and several biologically meaningful GO terms improved the prediction accuracy with GFBLUP for the four traits, as compared with GBLUP. The improvement of the genomic prediction between breeds (the average increase across the four traits was 0.161) was more apparent than that it was within the HOL (the average increase across the four traits was 0.020).

**Conclusions:**

Our genomic feature modelling approaches provide a framework to simultaneously explore the genetic architecture and genomic prediction of complex traits by taking advantage of independent biological knowledge.

**Electronic supplementary material:**

The online version of this article (doi:10.1186/s12864-017-4004-z) contains supplementary material, which is available to authorized users.

## Background

Studying the genetic architecture (e.g., the distribution of causal variants and their effects) and predicting future individual phenotypes for complex traits and diseases on the basis of genomic polymorphism data are very important in the fields of human medicine, adaptive evolution, and plant and animal breeding. Genomic predictions for such traits have been most often conducted by assuming that all of the genomic variants have a small effect drawn from the same prior distribution [1], such as in the standard genomic best linear unbiased prediction (GBLUP) and BayesA models [2]. As a result, the genomic variation of complex traits has always been treated as a “black box” that neither generates nor utilizes biological knowledge of the genetic architecture and the underlying biological mechanisms. This type of model performs well in populations with a large amount of LD (linkage disequilibrium), such as selectively bred plants and animals [3–5]. However, such models do not work well with populations of individuals not closely related, such as between breeds, probably because of differences in the segregated QTLs (quantitative trait loci), marker effects, allele frequencies and LD phases in such populations [3, 6]. For instance, the accuracy of the estimated genomic breeding values with GBLUP ranges from zero to very low in between-breed prediction in dairy cattle [3, 4, 7].

It has been proposed that shifting the focus from millions of whole genome sequence variants to those more likely to have functional effects might improve the accuracy of genomic predictions, especially in populations of not closely related individuals [8–12]. However, the genetic architecture of complex traits is currently poorly illustrated by single-marker genome-wide association studies (GWASs), owing to the many individually undetectable loci of small to moderate effects [13]. Therefore, the pre-selection of variants that might be causal on the basis of prior biological knowledge (e.g., Gene Ontology and pathway) may be key to improving prediction models, because it appears that the genomic variants associated with complex traits are more likely to be clustered in the genes belonging to biological pathways [9, 14, 15]. A secondary analysis of GWAS results (i.e., post-GWAS or marker set-test) based on biological priors may be a first step and a computationally simple way to explore the genetic and biological basis underlying complex traits [16]. Here, we also extended the standard GBLUP model by incorporating biological priors to implement this strategy, thus potentially leading to a better predictive ability of the model. This extended GBLUP model is called genomic feature BLUP (GFBLUP) model [9], and it includes an additional genomic effect that quantifies the joint effect on the trait of a group of variants located in a genomic feature. Both GBLUP and GFBLUP use all the genomic variants, but GFBLUP allows assignment of different weights to the genomic variants in each of the genomic relationships on the basis of their estimated genomic parameters, whereas GBLUP assumes that all of the genomic variants contribute to the determined genomic relationship equally. The GFBLUP model has previously been used to predict genetic values for complex traits in unrelated inbred lines of the *Drosophila melanogaster* Genetic Reference Panel (DGRP), and its prediction accuracy can be substantially improved by several Gene Ontology (GO) [17] terms that are enriched for causal genomic variants, as compared to the GBLUP model [9]. However, the GFBLUP model is much more computationally intensive compared to the post-GWAS analysis when evaluating many genomic features. Therefore, it could be important to investigate whether the post-GWAS analysis could be used to preselect the predictive genomic features, which can be used to develop more accurate GFBLUP models.

In this study, four complex traits (i.e.*,* milk, fat and protein yields, and mastitis) together with the imputed sequence variants in two dairy cattle breeds, Holstein (HOL, *n* = 5056) and Jersey (JER, *n* = 1231), were analysed. We hypothesized that the associated variants of these traits were likely to be clustered in genes belonging to GO terms of biological relevance and that this pattern might be consistent between breeds, although different breeds might have different mutations. The objectives of this study were 1) to explore the genetic and biological basis underlying milk production and mastitis by using post-GWAS analysis in the HOL training population (*n* = 4002), 2) to improve the prediction accuracy for these complex traits within and between breeds by using GFBLUP instead of GBLUP, and 3) to investigate the relationship between the degree of enrichment of association signals (i.e.*, P*-values) in a genomic feature based on post-GWAS in the HOL training population and its predictive ability with GFBLUP in the HOL validation population.

## Results

### Association signals of genomic variants from single-marker GWAS

Single-marker GWAS was separately conducted for milk production traits (i.e.*,* protein, milk and fat yields) and mastitis in a HOL training population using imputed sequence variants. The -log_10_(*P*) value of each tested variant for the four traits is shown in a Manhattan plot (Additional file 1: Fig. S1). The genomic inflation statistics (lambda) of the GWAS were less than 1.3 across the four traits, thus suggesting that the test statistics were not inflated by population stratification.

### Genomic feature classes

A total of 449 GO terms annotated for 4216 unique genes (~ 20% of all of the cattle Ensembl genes) were analysed. The average number of mapped variants in each of the studied GO terms was 2560 (ranging from 81 to 34,740). In total, the 449 GO terms could be grouped into 11 GO families (http://amigo.geneontology.org/amigo/dd_browse): immune system process (*n* = 12), response to stimulus (*n* = 66), cellular process (*n* = 50), localization (*n* = 40), behaviour (*n* = 4), metabolic process (*n* = 87), cellular component biogenesis (*n* = 32), developmental process (*n* = 62), biological regulation (*n* = 84), biological adhesion (*n* = 5), and reproduction (*n* = 7). The enrichment degree of the association signals in each of these GO families was compared between milk production and mastitis based on the post-GWAS analysis.

### Post-GWAS analysis helps to provide a genetic and biological understanding of milk production and mastitis

**Fig. 1:**
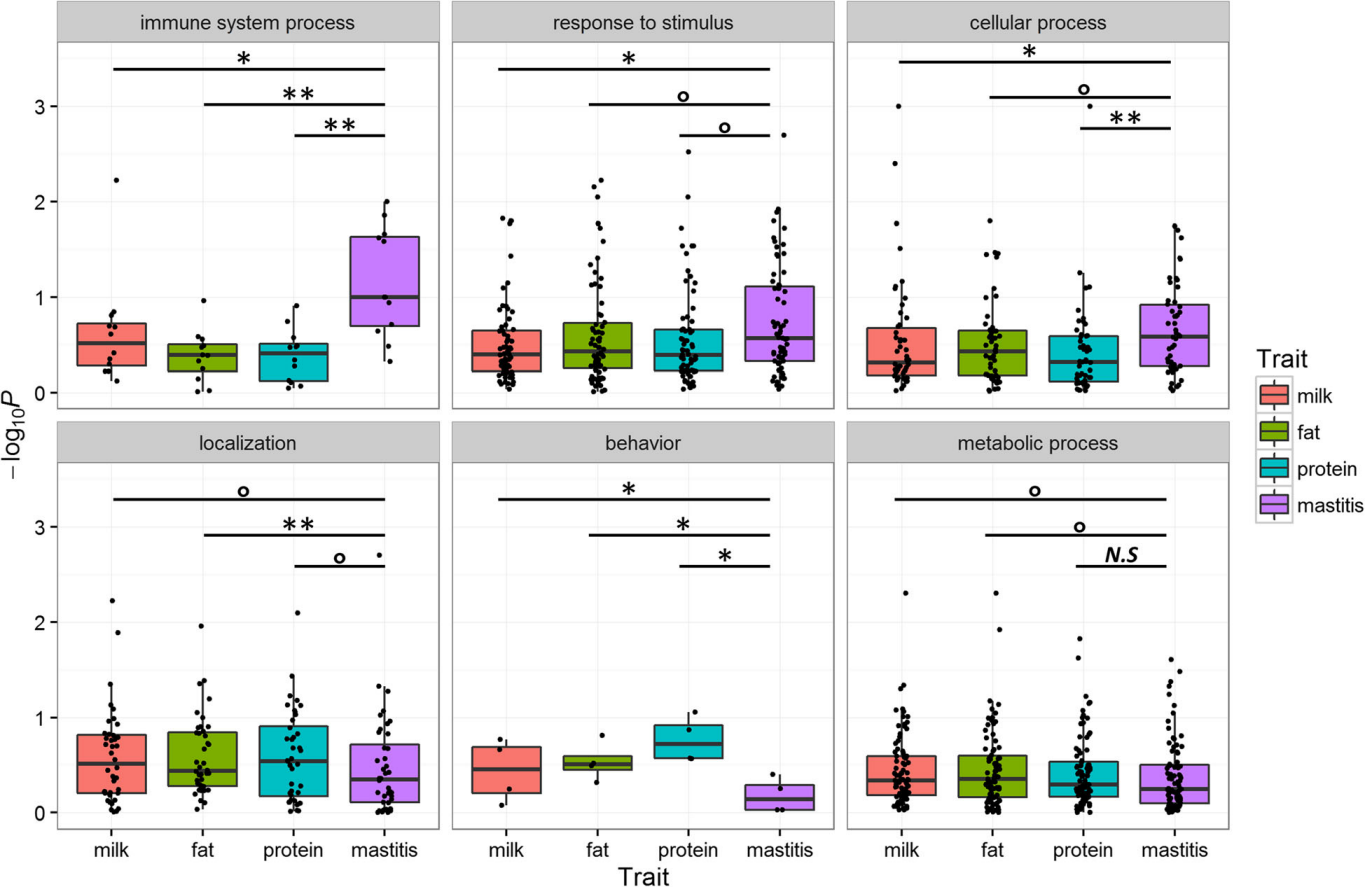
Comparisons of enrichment degrees of association signals between milk production and mastitis in Gene Ontology (GO) super-families in the Holstein (HOL) training population. Each point is a GO term. –log_10_ *P* is from post-GWAS analysis. The significant levels were determined on the basis of paired Student’s *t*-test: “**” means *P* < 0.01, “*” means *P* < 0.05, “о” means *P* ≤ 0.1, “*N.S*” means *P* ≥ 0.1

A post-GWAS analysis was conducted for each of the 449 GO terms in the four traits separately, on the basis of the GWAS results in the HOL training population. Detailed information on the post-GWAS analyses for the four traits is summarized in Additional file 2: Table S1, Additional file 3: Table S2, Additional file 4: Table S3 and Additional file 5: Table S4. As shown in Fig. 1, the enrichment degree of the association signals for mastitis had a tendency to be higher than that for milk production in the immune system process, response to stimulus, and cellular process, whereas the localization, behaviour, and metabolic process had a tendency to be more associated with milk production relative to mastitis. These findings indirectly provided supporting evidence that the genomic variants associated with milk production and mastitis were not randomly or uniformly distributed along the genome. This finding is not consistent with the assumption of infinitesimal models (e.g., GBLUP). The remaining GO super-families—cellular component biogenesis, developmental process, biological regulation, biological adhesion, and reproduction—did not show significant differences in the enrichment of the association signals between milk production and mastitis (Additional file 6: Fig. S2).

### GBLUP and GFBLUP analyses within the HOL breed

#### Improved prediction accuracy

**Fig. 2:**
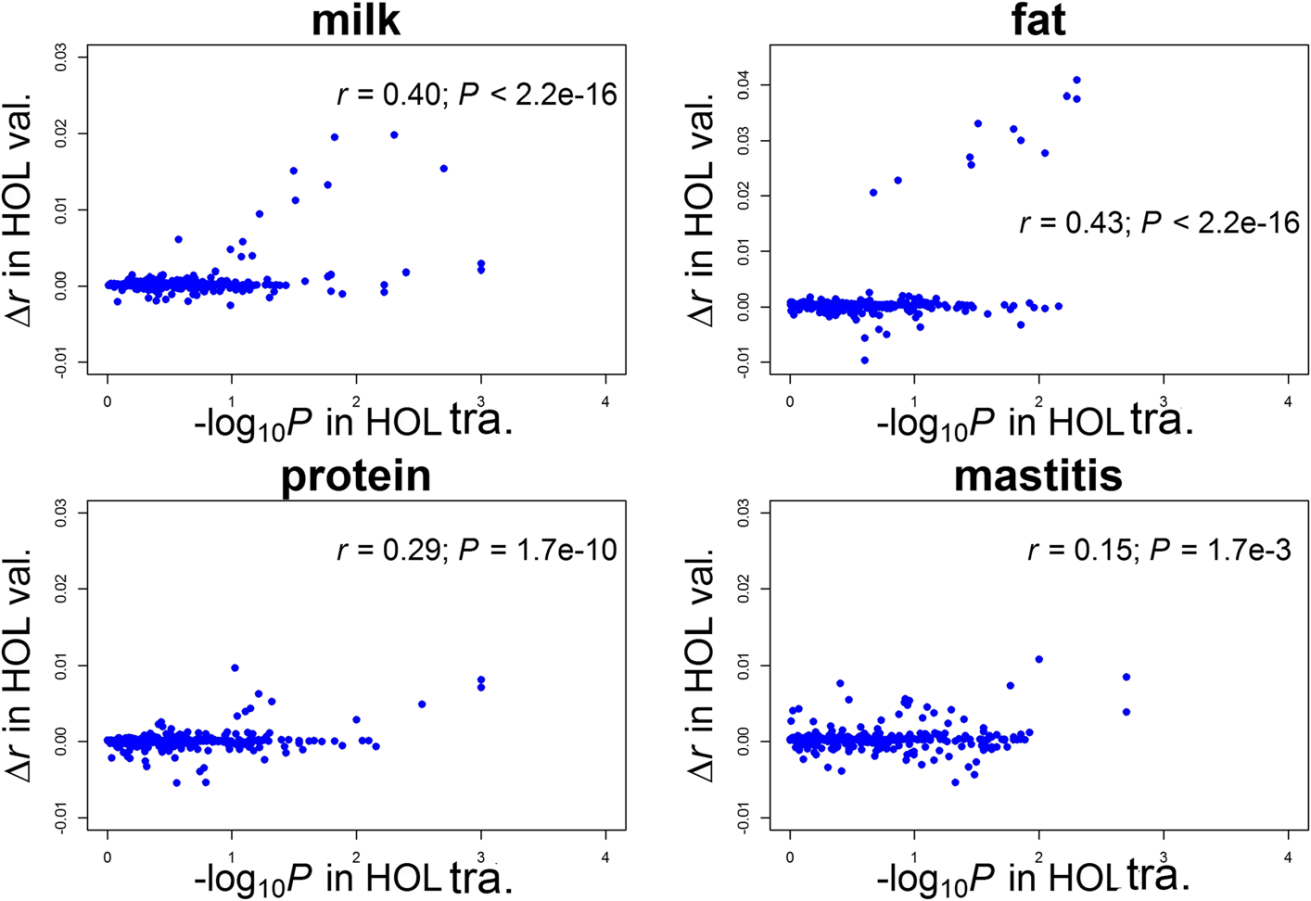
Comparisons of enrichment degree of association signals based on post-GWAS and the changes (∆*r*) in prediction accuracy with GFBLUP for all 449 Gene Ontology (GO) terms across the four traits. Each point is a GO term; −log_10_ *P* in the y axis is based on post-GWAS analysis in the HOL training population; *r* is the Pearson correlation, and *P* is determined with the correlation test

The prediction accuracy of the GBLUP model was 0.635 (bias = 0.862) for milk yield, 0.607 (bias = 0.808) for fat yield, 0.602 (bias = 0.775) for protein yield, and 0.504 (bias = 0.864) for mastitis. With the GFBLUP model, compared with the GBLUP model, 53, 64, 47, and 78 out of the 449 GO terms led to an increase of at least 0.001 in prediction accuracy for milk, fat and protein yields and mastitis, respectively, and these were considered predictive GO terms for each trait. Detailed information on the GFBLUP analyses for the four traits is summarized in Additional file 2: Table S1, Additional file 3: Table S2, Additional file 4: Table S3 and Additional file 5: Table S4. The changes in prediction accuracy with GFBLUP were significantly (*P* < 0.05) correlated with the degree of enrichment of association signals based on post-GWAS for all 449 GO terms across four traits (Fig. 2). These findings provided evidence that these predictive GO terms were not randomly detected from the GO database. The post-GWAS analysis in training population might be used to preselect predictive genomic features for GFBLUP models. However, some significant (*P* < 0.05) GO terms based on post-GWAS resulted in no or negative improvement in the accuracy of genomic prediction. Therefore, alternative post-GWAS methods should be developed to be better predictors of the genomic prediction improvement with GFBLUP. The top five predictive GO terms for each trait are summarized in Table 1. The average increase in prediction accuracy across the four traits with the best-performing GO term was 0.020. For the milk, fat and protein yields, the top predictive GO term was “retinol metabolic process”, with the increases of 0.020, 0.041 and 0.010 in prediction accuracy, respectively. Notably, the well-known milk-associated gene *DGAT1* was included in this GO term. Compared to GBLUP, several GO terms relevant to the immune response led to the increased prediction accuracies with GFBLUP for milk production traits, such as “response to lipopolysaccharides”, with the increases of 0.013 and 0.028 in prediction accuracy for milk and fat yield, respectively, and “defence response to bacteria”, with a increase of 0.006 in prediction accuracy for protein yield (Table 1). For mastitis, all of the top five predictive terms were engaged in immune responses, and the best-performing term was “positive regulation of activated T cell proliferation”, with an increase of 0.009 in prediction accuracy (Table 1). When the top five GO terms in each trait were combined as a single genomic feature, the prediction accuracy with GFBLUP was increased by 0.030, 0.046, 0.019 and 0.016 for milk yield, fat yield, protein yield and mastitis, respectively. In addition, when all GO terms in the “immune system process” were considered as a single genomic feature, the prediction accuracy with GFBLUP was increased by 0.012 for mastitis. These findings also provide biological insights into the genetic architecture underlying milk production and mastitis.

**Table 1:** Top five Gene Ontology (GO) terms with GFBLUP in Holstein for the four traits

Trait	GO ID	*r* ^a^ *_*_*GFBLUP*_	bias^b^	Δ*r* ^c^	${\left({H}_f^2\right)}^{\mathrm{d}}$	Nsets^e^	GO term	GO family
Milk	GO:0042572	0.655	0.863	0.020	0.169	586	Retinol metabolic process	Metabolic process
	GO:0034605	0.655	0.864	0.020	0.185	1517	Cellular response to heat	Response to stimulus
	GO:0040018	0.650	0.863	0.015	0.116	914	Positive regulation of multicellular organism growth	Biological regulation
	GO:0008285	0.650	0.865	0.015	0.209	4972	Negative regulation of cell proliferation	Biological regulation
	GO:0032496	0.648	0.864	0.013	0.144	1579	Response to lipopolysaccharides	Response to stimulus
Fat	GO:0042572	0.648	0.804	0.041	0.257	586	Retinol metabolic process	Metabolic process
	GO:0034605	0.645	0.804	0.038	0.291	1517	Cellular response to heat	Response to stimulus
	GO:0040018	0.644	0.801	0.037	0.200	914	Positive regulation of multicellular organism growth	Biological regulation
	GO:0007283	0.640	0.802	0.033	0.323	4273	Spermatogenesis	Reproduction
	GO:0000724	0.639	0.802	0.032	0.352	1308	Double-strand break repair via homologous recombination	Cellular process
Protein	GO:0042572	0.612	0.782	0.010	0.051	586	Retinol metabolic process	Metabolic process
	GO:0030154	0.610	0.783	0.008	0.016	9840	Cell differentiation	Developmental process
	GO:0090502	0.609	0.782	0.007	0.011	735	RNA phosphodiester bond hydrolysis, endonucleolytic	Cellular process
	GO:0042742	0.608	0.782	0.006	0.010	1231	Defence response to bacteria	Response to stimulus
	GO:0050821	0.607	0.781	0.005	0.021	3162	Protein stabilization	Biological regulation
Mastitis	GO:0042104	0.513	0.873	0.009	0.006	331	Positive regulation of activated T cell proliferation	Immune system process
	GO:0050729	0.513	0.872	0.009	0.007	626	Positive regulation of inflammatory response	Response to stimulus
	GO:0043066	0.512	0.871	0.008	0.047	8158	Negative regulation of apoptotic process	Biological regulation
	GO:0032465	0.511	0.872	0.007	0.014	151	Regulation of cytokinesis	Biological regulation
	GO:0006914	0.510	0.871	0.006	0.018	1753	Autophagy	Cellular process

^a^Prediction accuracy with GFBLUP

^b^The regression coefficient of de-regression proofs (DRP) on predicted genomic breeding values (GEBV)

^c^The change of prediction accuracy with GFBLUP relative to GBLUP

^d^Proportion of the total genomic variance explained by GO terms

^e^The number of SNPs in GO terms

#### Estimated genomic parameters

The total genomic heritabilities for GFBLUP (${h}_{GFBLUP}^2$) across all of the GO terms were very similar to those for GBLUP for all four traits (Additional file 2: Table S1, Additional file 3: Table S2, Additional file 4: Table S3 and Additional file 5: Table S4), thus indicating that the estimated genomic parameters with GFBLUP are not biased as compared with those with GBLUP. The proportions of the total genomic variance explained (${H}_f^2$) by the top five predictive GO terms were 11.6–20.9% for milk yield, 20.0–35.2% for fat yield, 1.0–5.1% for protein yield, and 0.6–4.7% for mastitis (Table 1). Notably, this range of total genomic variance was explained by only 0.003–0.029% of the total genomic markers (*SNP*_*f*_) for milk yield, 0.003–0.025% for fat yield, 0.003–0.057% for protein yield, and 0.001–0.047% for mastitis (Table 1). These findings provided further evidence that the genomic variance of these traits is not evenly or randomly distributed throughout the whole genome [15], but instead appears to be enriched in a subset of genomic regions defined by the GO terms. These findings further suggest that the genetic architecture of the studied traits is not consistent with the assumption of an infinitesimal model such as GBLUP.

### Improved prediction accuracy with GFBLUP between breeds by using predictive GO terms detected within the HOL breed

The prediction accuracies with the GBLUP model were very low when the entire HOL population (*n* = 5056) was used as a training set to validate JER individuals: 0.160 (bias = 0.762) for milk yield, 0.070 (bias = 0.482) for fat yield, 0.098 (bias = 0.622) for protein yield, and −0.058 (bias = −0.343) for mastitis. In total, 30 of the 53 predictive GO terms detected within the HOL breed were also identified as predictive (Δ*r* ≥ 0.001) between breeds for milk yield, 38 of 64 for fat yield, 29 of 47 for protein yield, and 46 of 78 for mastitis. Several GO terms led to decreases in the prediction accuracy with GFBLUP relative to GBLUP, probably because differently segregated QTLs or LD patterns between breeds led to “incorrect” weights being placed on the genomic variants in the features. The details of the GFBLUP analyses between breeds are summarized in Additional file 7: Table S5, Additional file 8: Table S6, Additional file 9: Table S7 and Additional file 10: Table S8. The improvement of the prediction with GFBLUP relative to GBLUP between breeds was more apparent than that within the HOL breed. The top five predictive GO terms for each trait between breeds are shown in Table 2. The average increase in prediction accuracy with the best-performing GO term was 0.161 across all four traits. For milk yield, the best-performing GO term was “positive regulation of multicellular organism growth”, with an increase of 0.200 in prediction accuracy. For fat yield, the best-performing term was “retinol metabolic process”, with an increase of 0.176 in prediction accuracy. For protein yield, the best-performing term was “defence response to bacteria”, with an increase of 0.134 in prediction accuracy. For mastitis, the best-performing term was “negative regulation of apoptotic process”, with an increase of 0.135 in prediction accuracy (Table 2). Notably, the GO term “response to lipopolysaccharides” led to an increase in prediction accuracy for both milk production and mastitis (Table 2), that is, 0.165 for milk yield, 0.130 for fat yield, and 0.125 for mastitis.

**Table 2:** Top five Gene Ontology (GO) terms with GFBLUP between breeds for the four traits

Trait	GO ID	*r* ^a^ *_*_*GFBLUP*_	bias^b^	Δ*r* ^c^	${\left({H}_f^2\right)}^{\mathrm{d}}$	Nsets^e^	GO term	GO family
Milk	GO:0040018	0.360	0.826	0.200	0.103	962	Positive regulation of multicellular organism growth	Biological regulation
	GO:0042572	0.342	0.808	0.182	0.171	678	Retinol metabolic process	Metabolic process
	GO:0034605	0.336	0.805	0.176	0.178	1621	Cellular response to heat	Response to stimulus
	GO:0045944	0.331	0.805	0.171	0.190	11,185	Positive regulation of transcription from RNA polymerase II promoter	Cellular process
	GO:0032496	0.325	0.798	0.165	0.129	1702	Response to lipopolysaccharides	Response to stimulus
Fat	GO:0042572	0.246	0.680	0.176	0.262	678	Retinol metabolic process	Metabolic process
	GO:0000122	0.238	0.642	0.168	0.348	8755	Negative regulation of transcription from RNA polymerase II promoter	Cellular process
	GO:0032496	0.200	0.577	0.130	0.219	1702	Response to lipopolysaccharides	Response to stimulus
	GO:0007283	0.176	0.538	0.106	0.313	4950	Spermatogenesis	Reproduction
	GO:0034605	0.171	0.558	0.101	0.271	1621	Cellular response to heat	Response to stimulus
Protein	GO:0042742	0.232	0.767	0.134	0.010	1333	Defence response to bacteria	Response to stimulus
	GO:0042475	0.224	0.732	0.126	0.011	3244	Odontogenesis of dentin-containing teeth	Developmental process
	GO:0006665	0.197	0.721	0.099	0.011	805	Sphingolipid metabolic process	Metabolic process
	GO:0042572	0.178	0.699	0.080	0.010	678	Retinol metabolic process	Metabolic process
	GO:0006810	0.168	0.693	0.070	0.040	6999	Transport	Localization
Mastitis	GO:0043066	0.077	0.277	0.135	0.064	8831	Negative regulation of apoptotic process	Biological regulation
	GO:0032496	0.067	0.176	0.125	0.020	1702	Response to lipopolysaccharides	Response to stimulus
	GO:0032091	0.045	0.171	0.103	0.032	702	Negative regulation of protein binding	Biological regulation
	GO:0043280	0.018	0.178	0.076	0.003	583	Positive regulation of cysteine-type endopeptidase activity involved in apoptotic process	Metabolic process
	GO:0071346	0.014	0.115	0.072	0.020	3494	Cellular response to interferon-gamma	Response to stimulus

^a^Prediction accuracy with GFBLUP

^b^The regression coefficient of de-regression proofs (DRP) on predicted genomic breeding values (GEBV)

^c^The change of prediction accuracy with GFBLUP relative to GBLUP

^d^Proportion of the total genomic variance explained by GO terms

^e^The number of SNPs in GO terms

## Discussion

To the best of our knowledge, few studies have simultaneously explored the genetic architecture and genomic prediction of complex traits in dairy cattle by integrating biological priors and whole sequence variants. Although the current GO annotation of the bovine genome (as observed in the current study only ~20% of genes were included) and the imputation accuracy of sequence genotypes are limited [16], our results still provided novel biological insights into the genetic architecture underlying milk production traits and mastitis and demonstrated that the prediction accuracy with GFBLUP can be improved over that with GBLUP by incorporating biological information of GO especially in between-breed prediction.

### GO terms associated with milk production and mastitis in dairy cattle

Here, we took the high-ranking predictive GO terms detected between breeds as examples of the power of our GFBLUP model to reveal biological processes associated with complex traits. For milk production, five GO terms, “positive regulation of multicellular organism growth”, “retinol metabolic process”, “response to lipopolysaccharides”, “positive regulation of transcription from RNA polymerase II promoter” and “cellular response to heat”, were highly predictive (Table 2). The first three GO terms have previously been proposed to be associated with milk production in studies on the cow mammary transcriptome during lactation cycles [18, 19]. For the latter two GO terms, “positive regulation of transcription from RNA polymerase II promoter” plays an important role in regulating the expression of genes [20] and the expression levels of many genes are altered during lactation [19], thus it may be interesting to investigate how “positive regulation of transcription from RNA polymerase II promoter” influences the milk production. Similarly, heat stress has been shown to directly affect feed intake, thus resulting in reduced milk production, especially in dairy breeds that generate substantial metabolic heat [21]. This result, together with our findings, provides supporting evidence that “cellular response to heat” may be associated with milk production traits.

For mastitis, all of the top five predictive GO terms, “negative regulation of apoptotic process”, “response to lipopolysaccharides”, “negative regulation of protein binding”, “positive regulation of cysteine-type endopeptidase activity involved in apoptotic process”, and “cellular response to interferon-gamma”, have previously been suggested to be associated with mastitis in transcriptome studies on specific tissues (e.g., liver and mammary gland) of cows with and without intra-mammary infection [22–25]. Of most interest is “response to lipopolysaccharides”, which is also highly predictive of milk production, consistently with results from a previous study [15] that partitioned the genomic variance of the milk production traits in HOL cattle by using the Kyoto Encyclopedia of Genes and Genomes (KEGG) pathways, thus revealing that several immune-relevant pathways (e.g., chemokine signalling pathway and leukocyte transendothelial migration) are significantly associated with milk production. All of these findings might reflect the genetic correlation between mastitis and milk production.

### Alternative biological priors

The genomic feature modelling approaches can be easily extended to integrate different sources of prior information, such as biological pathways, sequence ontologies, conservative genomic regions across species, and other types of evidence from functional experimental studies (e.g., transcriptomes and proteomes). The biological interpretation can become informative when additional layers of biological knowledge are included in the modelling approaches. However, a proportion of genes have not yet been functionally characterized or mapped to any manually curated or predicted pathways [16, 26–28], particularly in livestock and plants. Additionally, in this study, only approximately 10% of the total genomic variance in the milk yield was accounted for by its top predictive GO term, 26% for the fat yield, 1% for the protein yield, and 6% for masitits. Thus, further research on annotating the functional regions of the genome for a range of traits is required to realize the full potential of these genomic feature modelling approaches. Moreover, a host of functional modules (e.g., differentially expressed genes and differentially methylated regions) detected from independent experimental studies on same-scale populations may be used to develop more accurate genomic feature models for large-scale populations. Because increasing functional annotation data will be easily accessible for a range of traits and species, such as the ongoing Functional Annotation of Animal Genomes project (FAANG) [29], genomic feature modelling approaches should be increasingly useful.

### Post-GWAS with biological priors

Post-GWAS using prior biological knowledge may be a computationally simple approach to help open the “black box” of the genetic architecture underlying complex traits and to simultaneously offer novel insights into biological mechanisms. Multiple methods have been developed to implement this strategy, and the statistical properties of most of them have been thoroughly reviewed [30]. Our previous studies have shown that the performance of the current procedure is better than or similar to that of other commonly used methods (e.g., score or count -based) in most scenarios, especially when the following two criteria are met: 1) the average number of variants in each gene is approximately the same among the genomic features and 2) the average linkage disequilibrium (LD) between variants in different genes is approximately the same, so that the number of false positives can be very well controlled [12, 31].

### GFBLUP and alternative models

We hypothesize that the difference in prediction accuracy between GFBLUP and GBLUP is because the assumption of GBLUP (i.e., each genomic marker contributes to the genomic variance of the trait equally) does not match the genetic architecture of the traits. Instead, the genomic variants of complex traits seem to be enriched in certain genome regions. The genomic variants located in these enriched regions have greater weights than the remaining variants in GFBLUP, on the basis of their estimated genomic parameters, thus resulting in greater prediction accuracy. However, if the estimated genomic parameters deviate from their true values, less accurate predictions will result, because too much weight is placed on the “incorrect” genomic relationships in the prediction equations, as shown in Fig. 2, in which multiple GO terms lead to decreases in the prediction accuracy with GFBLUP relative to GBLUP. Previous simulation studies have demonstrated that the premise of the GFBLUP model is that genomic features are enriched in genomic variants associated with the traits and are less diluted by non-associated variants [9]. The imperfect imputation of whole-sequence variants may be another factor limiting the predictive ability of the GFBLUP model. All of the factors influencing the performance of GFBLUP have been discussed in detail previously [9, 12].

When the validation populations are very closely related to the training populations, the increase in prediction accuracy with GFBLUP may be limited compared to that achieved with GBLUP. In such populations with a high degree of LD, the determined genomic relationship in GBLUP (i.e., the individual genetic variants contribute to the genomic relationship equally) may provide accurate information about the causal genomic variants. A recent study has demonstrated that in a purebred Danish Duroc pig population, the increase in prediction accuracy with GFBLUP relative to GBLUP for complex traits (i.e., average daily gain, feed efficiency and lean meat percentage), incorporating prior information of QTL regions, ranges from very small to zero [12]. However, that simulation study has indicated that correctly separating the true causal variants from the non-causal (noise) markers in the GFBLUP further increases the prediction accuracy, even in such populations with highly related individuals [12]. Between breeds, the prediction accuracy with GBLUP for milk production and mastitis ranges from zero to very low, a result in agreement with findings from previous studies [3, 4, 7]. The GFBLUP models based on several GO terms have much greater increases in prediction accuracy compared with those within the HOL breed, thus reflecting that the GFBLUP model has the potential to improve genomic prediction between breeds, provided that they have similarities in the genetic architecture of the traits being investigated.

Our GFBLUP is implemented in a linear mixed-modelling framework, in which the known genetic and environmental factors can easily be adjusted [9, 32]. In addition, the genomic feature model was also implemented in Bayesian mixture models such as BayesRC [11]. The core element of the GFBLUP and GF Bayesian mixture models is the use of biological priors to partition the genomic variance. When sufficient information is available in the data, so that the data themselves can indicate which variants should have greater weights, the GF Bayesian mixture model might reliably assign the variants into the different variance classes defined in the model [9, 32], thus leading to a better prediction performance compared with that of GFBLUP. If this is not the case, a major difference between them in prediction performance is not expected [9, 32]. Compared with the GF Bayesian mixture model, GFBLUP is considerably more computationally efficient [11]. Compared with the post-GWAS approaches, both the GF Bayesian mixture models and GFBLUP are computationally intensive and require both the genotypes and phenotypes of the study populations.

## Conclusion

This study demonstrated that integrating prior biological knowledge on GO categories with whole-sequence variants can help to elucidate the genetic architecture and improve the genomic prediction of milk production and mastitis in dairy cattle, especially in between-breed prediction. The GFBLUP model is a flexible framework to simultaneously improve the understanding of the genetic architecture and the accuracy of the genomic prediction for complex traits, through taking advantage of independent biological priors, such as Gene Ontology and KEGG pathways. With the accumulation of biological knowledge regarding the functional annotation of the genome for a range of species, approaches such as GFBLUP will be increasingly useful, in particular for genomic prediction in validation populations that are not closely related to the training populations.

## Methods

### Phenotypes

The phenotypes used in this study were de-regressed proofs (DRP) of milk production traits (milk, fat and protein yields) and mastitis from a routine genetic evaluation by Nordic Cattle Genetic Evaluation (http://www.nordicebv.info/) and were available for 5056 HOL and 1231 JER cattle. All of the known fixed effects were corrected. Detailed information on these phenotypes has been previously described in [33–35]. The average reliabilities of the DRP for the milk, fat and protein yields and mastitis were 0.95, 0.95, 0.95, and 0.83, respectively, in HOL cattle and 0.92, 0.92, 0.92, and 0.76, respectively, in JER cattle. The heritability was 0.39, 0.39, 0.39, and 0.04 for milk, fat and protein yields and mastitis in HOL cattle and very similar in JER cattle [33, 35].

### Genotypes

Details of the imputation from the 50 K or High Density (HD) genotypes of these cattle to whole genome sequence data have been described previously [36, 37]. Briefly, the 50 K genotype for each individual was first imputed into a HD SNP array using IMPUTE2 v2.3.1 [38] on the basis of a multi-breed reference of 3383 animals (1222 HOL, 1326 Nordic red cattle, 835 JER) that had been genotyped using Illumina BovineHD chips (Illumina, Inc., San Diego, CA). A total of 648,219 SNPs were obtained after imputation to HD with an averaged accuracy of 0.97 [36]. The imputed HD genotypes were next imputed to the whole genome sequence level using *Minimac2* [39] on the basis of a multi-breed reference population of 1228 individuals from Run4 of the 1000 Bull Genomes Project [40] and additional whole genome sequences from Aarhus University including 368 HOL, 86 Nordic red, and 88 JER [41]. A total of 22,751,039 biallelic variants were obtained in the imputed sequence data, and the accuracy of imputation was 0.85 for 19,498,365 SNPs. Therefore, a given imputed sequence genotype (that was not in the 50 K) being correctly assigned was approximate 0.82 when considering the accuracy of the first step imputation (i.e.*,* from 50 k to HD) together. The details of the imputation accuracy were described in [37]. The imputed sequence dataset was further edited to exclude markers with a minor allele frequency (MAF) < 0.01 and a deviation from Hardy-Weinberg proportions (HWP) < 10^−6^. Finally, 15,355,382 and 13,403,916 SNPs remained for further analysis in HOL and JER cattle, respectively. It has been suggested this two-step imputation strategy is more accurate than the one-step strategy (i.e.*,* directly from 50 K to whole sequence) due to the complex LD pattern in dairy cattle, in particular when using individuals from multiple breeds as reference population [36].

### Training and validation populations

For the within-HOL prediction, the dataset was separated into training (*n* = 4002) and validation (*n* = 1054) sets on the basis of the animals’ birth years. The birth year cut-off was 2006, and the younger animals were assigned to the validation set. This validation strategy was chosen because it is the most meaningful in the context of dairy cattle breeding, in which young bulls are selected for breeding on the basis of their estimated genomic values, which are predicted using a training population of older animals with phenotypes. For the between-breed prediction, the entire HOL population (*n* = 5056) was used as training data to predict the genomic values of JER individuals (*n* = 1231).

### Genomic features

Genes grouped into a specific GO term were considered to be genomic features. The Bioconductor package “org.Bt.eg.db” v. 3.3.0 [42] was used to link genes to the GO terms. Here, we focused on only the GO terms belonging to “Biological processes”, and only the GO terms consisting of at least 10 directly evidenced genes were analysed. The imputed sequence variants were mapped to the bovine reference genome (UMD3.1). A genomic variant was assigned to a gene if the chromosome position of the variant was between the start and end chromosome positions of the gene (i.e.*,* within the open reading frames). Finally, a total of 615,329 genomic variants were linked to 4216 unique genes belonging to 449 GO terms.

### Sequence-based GWAS in the HOL training population

The association signals for the imputed sequence variants were assessed by using a two-step variance component-based method accounting for population stratification that was implemented in EMMAX [43]. The details of this model have been previously described [43]. In the first step, the polygenic and residual variances were estimated using the linear model

$$ \boldsymbol{y}=1\mu +\mathbf{Z}\boldsymbol{a}+\boldsymbol{e}, $$

where ***y*** is a vector of the phenotype (i.e., DRP); **1** is a vector of ones; *μ* is the overall mean; **Z** is a design matrix connecting phenotypes to random polygenic effects; ***α*** is a vector of random polygenic effects (i.e., breeding values), in which ***α*** ~ N(**0**, $\mathbf{G}{\sigma}_a^2$), and **G** is the genome relationship matrix built using HD genotypes, excluding the chromosome harbouring the candidate SNP for controlling double fitting (i.e., fitting the variant as a random effect as part of the **G** and a fixed effect for testing association) [44], and ${\sigma}_a^2$ is the additive genetic variance; and ***e*** is the vector of residuals, where ***e*** ~N(**0**, $\mathbf{I}{\sigma}_e^2$), and **I** is the identity matrix, and ${\sigma}_e^2$ is the residual variance. In the following step, the individual variant effect was assessed using the linear regression model

$$ \boldsymbol{y}=1\boldsymbol{\mu} +\boldsymbol{xb}+\boldsymbol{\eta}, $$

where ***y*** and **1** are the same as described above, ***x*** is a vector of genotype dosages (ranging from 0 to 2), ***b*** is the allele substitution effect (i.e., variant effect), and ***η*** is a vector of random residual deviates with (co)variance structure $\mathbf{G}{\sigma}_a^2+\mathbf{I}{\sigma}_e^2$ . The genome-wide significance thresholds corresponding to an error rate of 0.05 were set at 3.3 × 10^−9^, on the basis of Bonferroni multiple testing correction. Manhattan plots were generated using *qqman* v.0.1.2 in the R package [45]. The genomic inflation statistic (lambda) was defined as the median of the resulting chi-squared test statistics divided by the expected median of the chi-squared distribution with one degree of freedom.

### Post-GWAS analysis in the HOL training population

Because the genomic variance of the milk production and mastitis has been generally considered to be governed by many genes, each having small to moderate effects, the following summary test statistic of a genomic feature (i.e., a GO term) was used, which may be more powerful than the commonly used count-based methods described previously [12, 31]:

$$ {T}_{sum}={\sum}_{i=1}^{mf}{t}^2, $$

where m_f_ is the number of variants located in a genomic feature, and *t* ^2^ is the square of *t*, which was calculated as the estimated effect of a variant divided by its standard error. The cyclical permutation strategy applied to test the association between a phenotype and a genomic feature was described previously [12, 31]. Briefly, the observed test statistic (i.e., *t* ^2^) of each variant was ranked according to the chromosome position of the variant (i.e.*,* *t*_1_, *t*_2_ ⋯ *t*_*t*−1_, *t*_*t*_). A test statistic (i.e.*,* *t*_k_) was randomly chosen from this vector. All test statistics were then shifted to the new positions, where the selected one (i.e.*,* *t*_k_) became the first, and the statistics of the other variants were shifted to new positions, but retained their original order (i.e.*,* *t*_k_, *t*_k+1_ ⋯ *t*_*t*_, *t*_1_ ⋯ *t*_k-1_). Any association between the variants and genomic features was uncoupled while maintaining the correlation structure among test statistics. Afterward, a new summary statistic of a genomic feature was calculated according to the original chromosome position of the feature. This permutation was repeated 1000 times for each tested genomic feature, and an empirical *P*-value was calculated on the basis of one-tailed tests of the proportion of randomly sampled summary statistics larger than that observed.

### Genomic prediction models

For each of the 449 genomic features, a separate analysis was conducted. By partitioning the genomic variants into two sets (within the genomic feature and the remaining genome), in each of the GFBLUP analyses, the collective contribution of a genomic feature to the trait was evaluated. The GFBLUP model is

$$ \boldsymbol{y}=1\mu +{\boldsymbol{g}}_{\boldsymbol{f}}+{\boldsymbol{g}}_{\boldsymbol{R}}+\boldsymbol{e}, $$

where ***y*** is the vector of phenotypic observations (i.e., DRP), **1** is a vector of ones, μ is the overall mean, ***g***_***f***_ is the vector of genetic values captured by variants in the genomic feature, ***g***_***R***_ is the vector of genomic values captured by variants in the remaining genome, and ***e*** is the vector of residuals. The assumptions for all of the random effects are given by

$$ \left(\begin{array}{c}{\boldsymbol{g}}_{\boldsymbol{f}}\\ {}\begin{array}{c}{\boldsymbol{g}}_{\boldsymbol{R}}\\ {}\boldsymbol{e}\end{array}\end{array}\right)\sim N\left[\left(\begin{array}{c}0\\ {}\begin{array}{c}0\\ {}0\end{array}\end{array}\right),\left(\begin{array}{cc}\begin{array}{cc}{\boldsymbol{G}}_{\boldsymbol{f}}{\sigma}_f^2& 0\\ {}0& {\boldsymbol{G}}_{\boldsymbol{R}}{\sigma}_R^2\end{array}& \begin{array}{c}0\\ {}0\end{array}\\ {}0\kern2.50em 0& \boldsymbol{D}{\sigma}_e^2\end{array}\right)\right] $$,

***G***_***f***_ and ***G***_***R***_ are genomic relationship matrices, built using the variants in the genomic feature and the remaining genome, respectively. Both ***G*** were calculated using the second method described by VanRaden (2008) [46]. ***D*** is a diagonal matrix with diagonal elements equal to $\frac{1-{r}^2}{r^2}$, where *r* ^2^ is the reliability of a DRP. ${\sigma}_f^2$ and ${\sigma}_R^2$ are the variance components accounted for by the variants in the genomic feature and the remaining genome, respectively, and ${\sigma}_e^2$ is a residual variance component. All of these variance components were estimated using an average information restricted maximum likelihood (REML) procedure [47], as implemented in DMU [48].

The proportion of the genomic variance explained by the genomic feature was calculated as

$$ {H}_f^2=\frac{\sigma_f^2}{\sigma_f^2+{\sigma}_R^2} $$,

The proportion of SNPs in the genomic feature was calculated as

$$ {SNP}_f=\frac{m_f}{m_f+{m}_R} $$,

where *m*_*f*_ is the number of variants in the genomic feature, and *m*_*R*_ is the number of variants in the remaining genome.

GBLUP uses only one random genomic effect,

***y*** = 1*μ* + ***g*** + ***e***,

with the same notation as above except for ***g***, which is the vector of genomic values captured by all of the genomic variants. The random genomic values and the residuals were assumed to be independently distributed: $\sim N\left(0,\boldsymbol{G}{\sigma}_g^2\right)$ and $\boldsymbol{e}\sim N\left(0,\boldsymbol{D}{\sigma}_e^2\right)$.

Inferences on the genomic heritability for GFBLUP and GBLUP were calculated as

${h}_{GFBLUP}^2=\frac{\sigma_f^2+{\sigma}_R^2}{\sigma_f^2+{\sigma}_R^2+{\sigma}_e^2}$ for GFBLUP, and ${h}_{GBLUP}^2=\frac{\sigma_g^2}{\sigma_g^2+{\sigma}_e^2}$ for GBLUP

**Genomic prediction accuracy:** In the GFBLUP model, the total genomic value (GEBV) is ${\widehat{\boldsymbol{g}}}_{total}={\widehat{\boldsymbol{g}}}_{\boldsymbol{f}}+{\widehat{\boldsymbol{g}}}_{\boldsymbol{R}}$, and in GBLUP it is ${\widehat{\boldsymbol{g}}}_{total}=\widehat{\boldsymbol{g}}$. The accuracy of the predicted genomic breeding values (*r*) is calculated as the correlation between GEBV and DRP in the validation populations. The bias of genomic predictions was measured as the regression coefficient of DRP on the GEBV, i.e. $\mathrm{bias}=\operatorname{cov}\left(\mathrm{DRP},\mathrm{GEBV}\right)/{\upsigma}_{\mathrm{GEBV}}^2$.

## Additional files

Additional file 1: Fig. S1. Manhattan plots of sequence-based genome-wide association analyses in the Holstein (HOL) training population. (TIFF 602 kb)

Additional file 2: Table S1. Results of Post-GWAS and GFBLUP analyses for milk yield within the Holstein (HOL) breed. (XLSX 82 kb)

Additional file 3: Table S2. Results of Post-GWAS and GFBLUP analyses for fat yield within the Holstein (HOL) breed. (XLSX 60 kb)

Additional file 4: Table S3. Results of Post-GWAS and GFBLUP analyses for protein yield within the Holstein (HOL) breed. (XLSX 60 kb)

Additional file 5: Table S4. Results of Post-GWAS and GFBLUP analyses for mastitis within the Holstein (HOL) breed. (XLSX 119 kb)

Additional file 6: Fig. S2. Comparisons of enrichment degrees of association signals in the remaining Gene Ontology (GO) super-families between milk production and mastitis in the Holstein (HOL) training population. Each point is a GO term. –log_10_ *P* is from post-GWAS analysis. The significant levels were determined with paired Student’s *t*-test. The significance levels of the comparisons are not shown, as *P* ≥ 0.1. (TIFF 149 kb)

Additional file 7: Table S5. Results of GFBLUP analyses for milk yield between Holstein (HOL) and Jersey (JER) breeds. (XLSX 32 kb)

Additional file 8: Table S6. Results of GFBLUP analyses for fat yield between Holstein (HOL) and Jersey (JER) breeds. (XLSX 33 kb)

Additional file 9:: Table S7. Results of GFBLUP analyses for protein yield between Holstein (HOL) and Jersey (JER) breeds. (XLSX 32 kb)

Additional file 10: Table S8. Results of GFBLUP analyses for mastitis between Holstein (HOL) and Jersey (JER) breeds. (XLSX 34 kb)

## Abbreviations

GBLUP: Genomic best linear unbiased prediction
GFBLUP: Genomic feature best linear unbiased prediction
GO: Gene Ontology
GWAS: Genome-wide association study
HOL: Holstein
JER: Jersey
LD: Linkage disequilibrium
MAF: Minor allele frequency
