# Supplementary figures and images for: Use of biological priors enhances understanding of genetic architecture and genomic prediction of complex traits within and between dairy cattle breeds
Source: BMC Genomics. 2017 Aug 10;18:604. doi: 10.1186/s12864-017-4004-z (PMC5553760; doi:10.1186/s12864-017-4004-z)

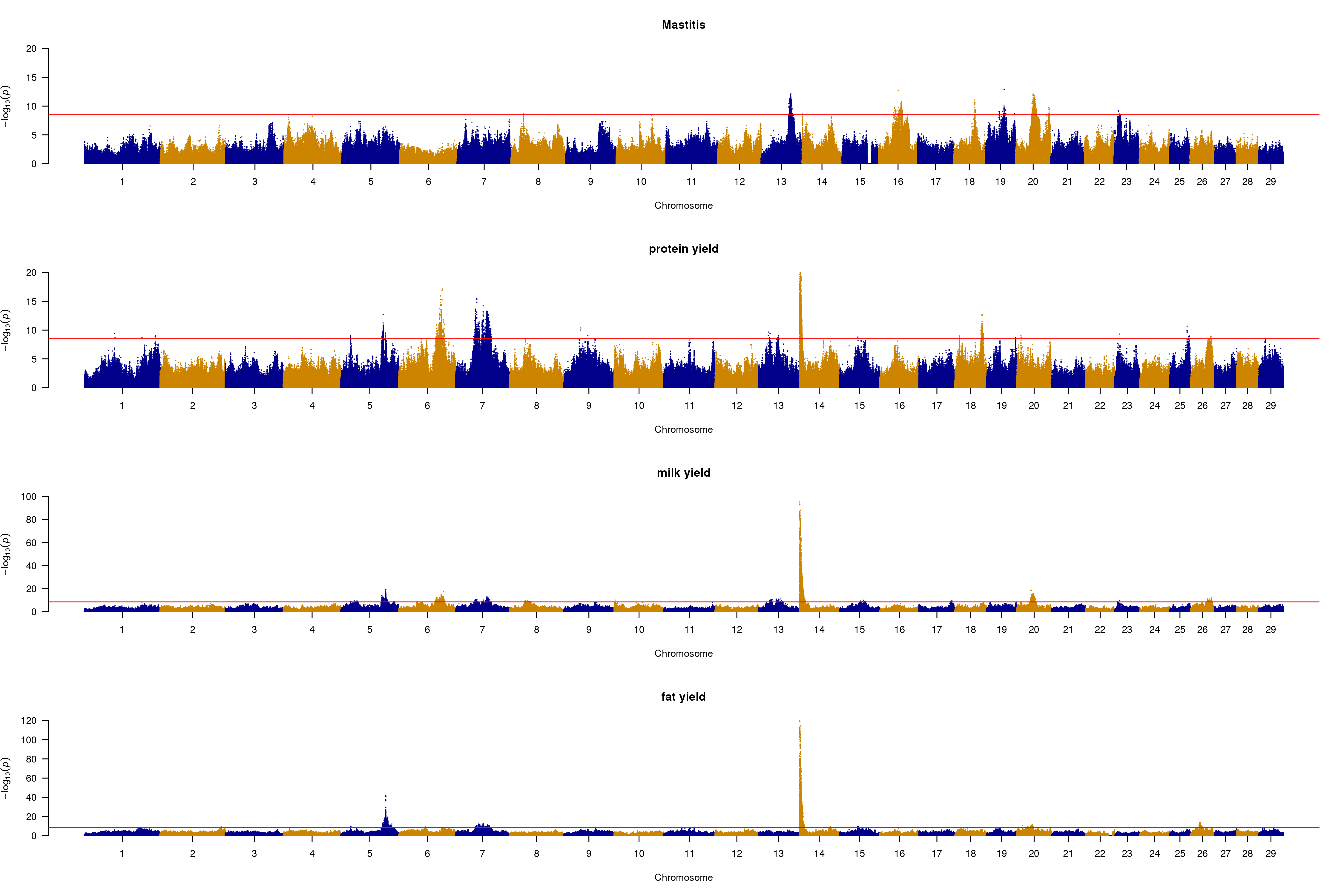

Supplement: Supplementary file 1 — Manhattan plots of sequence-based genome-wide association analyses in the Holstein (HOL) training population. (TIFF 602 kb) [file 12864_2017_4004_MOESM1_ESM.tiff]

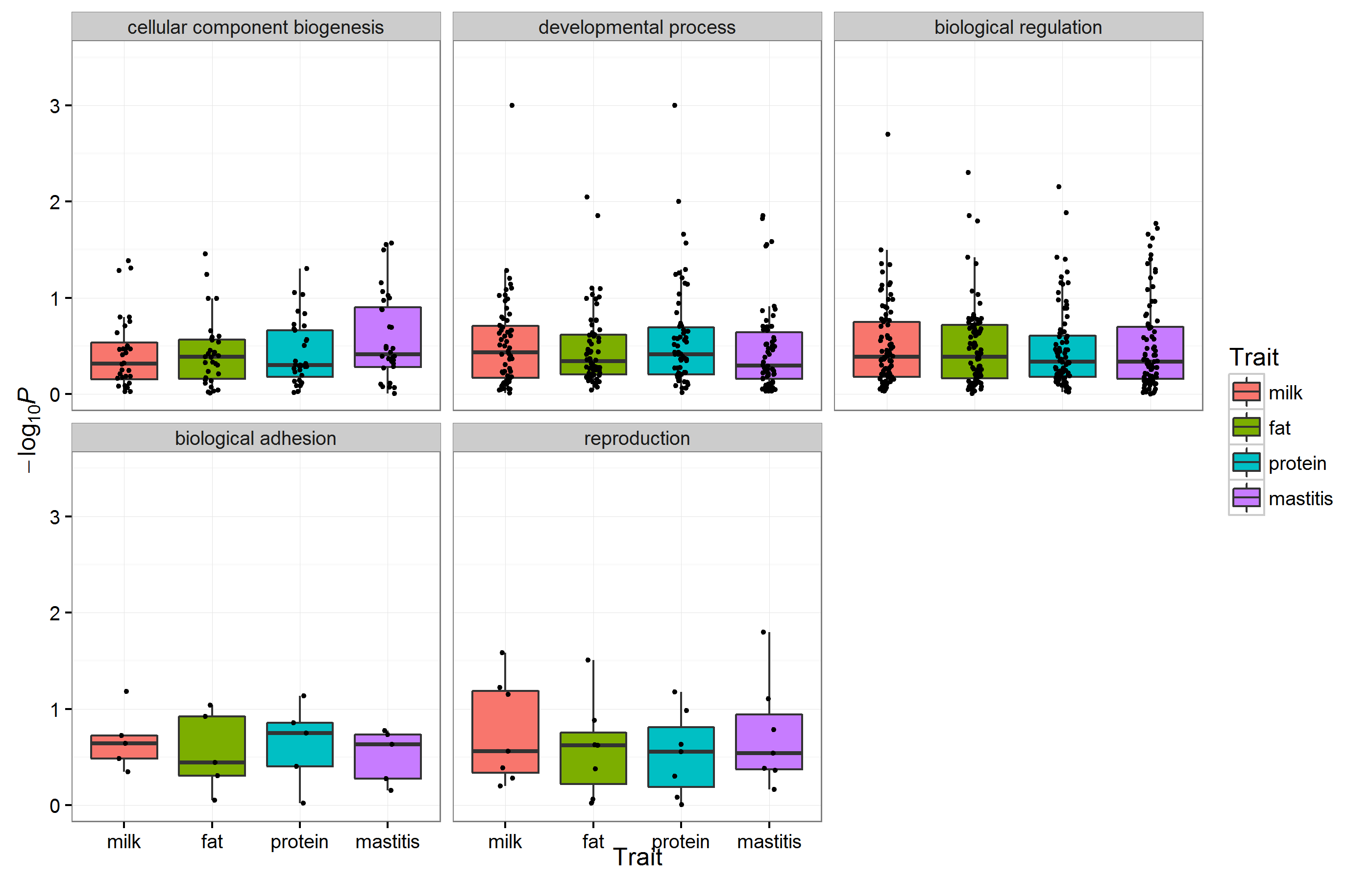

Supplement: Supplementary file 6 — Comparisons of enrichment degrees of association signals in the remaining Gene Ontology (GO) super-families between milk production and mastitis in the Holstein (HOL) training population. Each point is a GO term. –log10 P is from post-GWAS analysis. The significant levels were determined with paired Student’s t-test. The significance levels of the comparisons are not shown, as P ≥ 0.1. (TIFF 149 kb) [file 12864_2017_4004_MOESM6_ESM.tiff]
